# The relationship between client dissatisfaction and contraceptive discontinuation among urban family planning clients in three sub-Saharan African countries

**DOI:** 10.1371/journal.pone.0271911

**Published:** 2022-08-22

**Authors:** Carolina Cardona, Funmilola M. OlaOlorun, Elizabeth Omulabi, Peter Gichangi, Mary Thiogo, Amy Tsui, Philip Anglewicz

**Affiliations:** 1 Department of Population, Family, and Reproductive Health, Bloomberg School of Public Health, Johns Hopkins University, Baltimore, Maryland, United States of America; 2 Institute for Advanced Development Studies, La Paz, Bolivia; 3 Department of Community Medicine, University of Ibadan, Ibadan, Oyo State, Nigeria; 4 Department of Statistics and Population Studies, University of the Western Cape, Bellville, South Africa; 5 International Center for Reproductive Health, Nairobi, Kenya; 6 Department of Public Health and Primary Care, Faculty of Medicine and Health Sciences, Ghent University, Gent, Belgium; Jhpiego, UNITED STATES

## Abstract

Although researchers and practitioners have suggested that the quality of family planning services impacts contraceptive discontinuation, establishing a causal relationship has been challenging, primarily due to data limitations and a lack of agreement on how to measure quality. This longitudinal study estimated the relationship of the dissatisfaction with family planning services on contraceptive discontinuation for a sample of 797 female clients who sought family planning services at urban facilities across Kenya, Nigeria, and Burkina Faso. Clients who sought family planning services were first interviewed in person at private and public health facilities and received a follow-up phone interview four to six months later. In our sample, 18.2% of clients who were using a modern contraceptive at baseline stopped using it by follow-up. At baseline, nearly 14% of clients reported experiencing a problem with service convenience, nearly 12% with the availability of medicines and contraceptives, and nearly 6% with facility cleanliness and/or staff treatment. We hypothesized that client dissatisfaction with the family planning services received informed their decision to discontinue contraception and estimated univariate and bivariate probit regression models, controlling for individual and health facility characteristics. We found that client’s perceptions of staff treatment and facility cleanliness informed their expectations about service and contraceptive standards, affecting subsequent contraceptive discontinuation. The difference in the probability of discontinuing contraception was 8.2 percentage-points between dissatisfied and satisfied clients. Examining client dissatisfaction with family planning services can inform the family planning community on needed improvements to increase contraceptive adherence for women in need, which can prevent unplanned pregnancies and unwanted births in the long run.

## 1. Introduction

In developing countries, 44% of pregnancies were unintended between 2010 and 2014, almost two-thirds of which ended up in abortion, putting the health of mothers at risk where abortion is illegal or limited and unsafe [1]. Contraceptive use and continuation is an optimal solution to reduce the number of unplanned pregnancies and unintended births: in 36 developing countries between 2009 and 2014, contraceptive discontinuation was responsible for about one-third of unintended births [2]. A study of 19 countries from Africa, Asia, Eastern Europe, and Latin America, estimated that on average, 38% of women who were contraceptive users discontinued within the first year, and 55% by the second year [3].

Contraceptive discontinuation is a dynamic measure of contraceptive use; it reflects the contraceptive status change from user to nonuser for women who want to delay or avoid pregnancy. Like other dynamic measures such as contraceptive adoption and method switching, it would be ideal to follow women throughout their reproductive careers to measure changes in contraceptive use over time. However, this is infrequently done; research from developing countries tends to rely instead on the contraceptive calendar produced by the Demographic and Health Survey (DHS) Program [2–9], which asks women to recall their monthly contraceptive use during the five years preceding the interview. Users’ reasons for ending the use of a method will vary from voluntarily stopping to have a desired pregnancy to accidental pregnancy, to involuntarily terminating due to concern about health effects or partner disapproval [3, 6].

A study of contraceptive dynamics helps to understand women’s movements across contraceptive use categories, and reasons for these movements. Although the contraceptive calendar is a useful tool for studying contraceptive dynamics, there are several limitations. First, the calendar suffers from recall bias affecting the quality of the data. Prior assessments have found the calendar typically underestimates contraceptive use [10], and that it is more reliable for long-acting than for short-acting contraceptive methods [11]. Second, although the DHS calendar captures contraceptive use for five years prior to the survey, other factors associated with contraceptive dynamics are only measured at the time of the survey (e.g., socioeconomic status, education, number of children, fertility preferences). These characteristics captured at the time of the interview may have been altered by prior contraceptive behavior.

Individual factors, such as age, education, parity, community-level contraceptive use, fertility preferences, and method-related problems are contributing factors of contraceptive discontinuation [3, 7, 12, 13]. However, most of these findings in the published research literature used retrospective data where individual factors are measured at the time of the interview, and not necessarily over the time leading up to discontinuation. Studies that use longitudinal prospective data confirm the time-varying nature of covariates’ effects which reduce need and method-related problems for discontinuation, but there are relatively few such studies [13, 14]. Our understanding of the determinants of contraceptive discontinuation will improve with the expanded availability of longitudinal data.

Beyond individual-level determinants, the health system and care environments exercise their own influences on clients’ contraceptive use [15]. Most household or population survey data sources, however, are not structured to enable linkage with patient care data to reveal supply-side dynamics, such as contraceptive stock availability and provider-patient interactions. The few studies that have investigated the association between health services and women’s contraceptive discontinuation in developing countries have found that providing method-related information has the potential of increasing continuation [13, 16]. Feeser et al. (2019) found in a sample from urban Kenya that the rate of continuation among women receiving family planning information versus receiving none was 50% higher; privacy, autonomy, and dignity were also significant service quality factors. Similarly, evidence from high-income countries suggests that healthcare providers play an important role in maintaining use [17] or encouraging women to continue using contraception when they do not want to have a child [18]. Even if women switch methods, providers can support them with a smooth transition to another method that satisfies their needs.

Research shows that the physician-patient relationship and patients’ satisfaction with their healthcare providers impact patients’ adherence to treatment [19, 20]. Poor physician-patient relationships may also affect women’s decision to stop using contraception. Dehlendorf et al. (2016) found that high quality interpersonal counseling can sustain patients continued use of contraception six months later. In addition, Bruce (1990) and Jain (1989) both hypothesized that the patient-provider relation is a key element in the service environment that impacts contraceptive acceptance and continuation in low-income settings.

Elements of the quality-of-care framework that were proposed by Bruce (1990) and Jain (1989) have been investigated across different settings, yet no single standardized metric has been agreed upon. However, studies have found that method selection, respectful care, and effective use of method selected were reflective of quality of care in the provision of family planning services and influenced women’s decision to discontinue their use of contraception (e.g., Jain et al. 2019, RamaRao et al. 2003).

Using data from female urban clients sampled from health facilities in Kenya, Nigeria, and Burkina Faso, this study aims to measure the effect of client dissatisfaction with family planning services on the risk of contraceptive discontinuation. Our study design is distinct because we can link each family planning client to the urban health facility where she obtained contraceptive supplies or services. We measure each client’s dissatisfaction with the facility’s services and other selected facility features present at the time when she was prescribed or dispensed a method of contraception. We then follow her continued use of that contraceptive method four to six months later. Examining the influences of client dissatisfaction with service conditions on contraceptive discontinuation can inform health service researchers of their relative importance for contraceptive adherence, which in the long run can prevent clients’ unplanned pregnancies and unwanted births.

## 2. Materials and methods

### 2.1. Data

Data for this study are drawn from the Performance Monitoring for Action (PMA) Agile project, a continuous data monitoring and evaluation system designed to inform family planning program managers. PMA Agile facility surveys were implemented quarterly in a probability sample of health facilities in eight urban sites in Kenya, Nigeria, and Burkina Faso following a similar sampling protocol. The study began with a full list of public and private family planning service delivery points in each urban area and then randomly selected 220 facilities in each site, with roughly equal numbers being public or private. More information about PMA Agile can be found at the project’s website: https://www.pmadata.org/technical-areas/pma-agile and in the PMA Agile study protocol paper [21].

PMA Agile’s Service Delivery Point (SDP) questionnaires were mainly completed by the in-charge/manager of the health facility, although other facility personnel could contribute information on contraceptive stock supplies and equipment. The person who responded to the SDP questionnaire was informed about the PMA Agile project and provided its written consent to begin the interview. The SDP questionnaire focused on the provision of family planning methods and services, stock status, fees charged, client volume, and service integration.

For this study, we also used data from the Client Exit Interview (CEI) surveys conducted with two prospective cohorts of female clients of reproductive age (18–49) enrolled and interviewed during the quarterly facility survey either in 2018 or 2019 and followed up four to six months later by phone. Table A-1 in S1 Appendix provides the dates when data for each cohort were collected by country. The first interview was conducted in person at the health facility where the client received family planning services. At the beginning of the interview, the client received information about the PMA Agile project and provided its written consent to begin the interview. A mobile airtime card with a value of one USD was provided to each enrolled client completing the interview. At the end of the CEI, female clients were asked if they would be willing to be re-interviewed by telephone after approximately four to six months and, if so, to provide up to two telephone numbers. The proportion of clients who provided a telephone number was 86.5% in Kenya, 60.4% in Nigeria, and 96.3% in Burkina Faso. Overall, 88.9% of baseline clients in Kenya completed the follow-up interview, 61.9% in Nigeria, and 79.3% in Burkina Faso. The same interviewer at baseline generally conducted the follow-up interview. Mobile phone airtime equivalent to one USD was again transmitted electronically upon completion of the follow-up interview. The CEI survey instrument included questions on social and demographic characteristics, contraceptive use, service quality, and family planning product recognition. Clients were also asked about current, continued, and future contraceptive use, method switching, and exposure to public messaging on family planning.

Although both female and male clients who presented at the health facility for general and reproductive health services were recruited to participate in the CEIs, we restrict our analytic sample to female clients receiving family planning services at baseline in order to take advantage of the information on facility services present at that time. This restriction led to 1,009 female family planning clients retained out of a total of 15,305 female clients. The method of contraception recorded at baseline was the method clients received or was prescribed on the day of the interview (795 clients). There were 214 clients who were neither prescribed nor dispensed contraception during their visit. These clients were asked to indicate the method they were using at that time. We excluded baseline users of emergency contraception and sterilization, reducing the sample to 973. We did not include clients who in the follow-up survey reported a different method of contraception than the one reported in the baseline survey because we cannot know whether they actually changed methods or it is a misreport of their method [22]. This restriction dropped 176 clients, yielding a sample of 797 clients of whom 765 had complete CEI and SDP information. The baseline composition of methods used by the client samples for each of the three countries was similar, see Fig A-1 in S1 Appendix, which provides assurance for combining them for a pooled analysis.

We did not seek ethical approval for this analysis of de-identified secondary data. Data collection protocols by the PMA Agile project were reviewed and approved by the institutional review board of the Johns Hopkins Bloomberg School of Public Health, and the in-country counterpart institutional review boards. For more information see the Ethical approval section in Tsui, A. et al. (2020).

### 2.2. Measures

All women included in our sample were using contraception at the baseline interview. At the follow-up survey, they had three alternatives: i) to stop using contraception; ii) to continue using the same method of contraception (than the one reported at baseline); and iii) to switch to a different method of contraception (than the one reported at baseline). We did not include switchers in this study. The first outcome of interest, contraceptive discontinuation, was constructed as a binary measure based on the client’s report of the baseline method and the contraceptive use reported at the time of the follow-up interview. The measure took the value of one if the client reported using a modern contraceptive method at baseline and subsequently reported it was no longer using contraception, and zero if the client was using the same baseline method at follow-up.

For the second outcome, clients’ dissatisfaction with family planning services, we created four binary measures from baseline CEI data. We assume that clients’ interactions with healthcare providers at the time they received their contraceptive method will inform their subsequent contraceptive behaviors by shaping more immediate expectations about service and contraceptive performance. These four measures are based on the clients’ dissatisfaction with twelve family planning service conditions. Respondents were asked “Now I am going to ask about some common problems clients have at health facilities. As I mention each one, please tell me whether any of these were problems for you today, and if so, whether they were major or minor problems for you”. The list of potential problems included: 1) time waited to see a provider; 2) amount of explanation received about the problem or treatment; 3) visual privacy during the examination; 4) auditory privacy during the consultation; 5) range of services available; 6) availability of medicines; 7) availability of contraceptives; 8) hours of service (i.e., when they open and close); 9) number of days services are available; 10) cleanliness of the facility; 11) how treated by staff (This question asked specifically: How the staff treated you.); and 12) cost of services or treatment. As interviewers mentioned each one of the problems, clients indicated whether any of these were not a problem, a minor problem, or a major problem for them during their visit. These variables were transformed into binary form, for which experiencing minor or major problems were grouped together as problematic. A principal component analysis, described below, was then conducted to identify the four components of client dissatisfaction with family planning services.

The baseline covariates of interest used to adjust the estimated effects were grouped in two categories: service delivery point measures (managing authority (private or public), in-stock status of long-and short-term contraceptive methods, fees charged for family planning services, and discussing family planning with the provider) and individual characteristics (prior use of the baseline method, baseline method, exposure to family planning through radio or TV in past three months, discussing family planning with a community health worker, marital status, age, parity, education, household economic status, and partner communication about family planning in the last six months). These covariates were selected according to those that previous research has found to be associated with contraceptive discontinuation, as discussed in the background section. Method stocks were categorized separately as long-term or short-term methods in binary form that equals one if the facility had any method out-of-stock or if they were not offered, and zero otherwise. Client age was grouped into three categories, 18–24, 25–34, and 35–49 years. Parity level was categorized as 0–1, 2–3, and 4 or more children. Education level was categorized as None/Primary, Post-primary/Secondary, and College/University. Household economic status was measured using the Cantril [23] ladder, in which clients ranked their household well-being on a 10-step staircase where the first step represents the poorest, and the 10th step represents the richest [24]. Household economic well-being was grouped into poorest (1–3), middle (4–5), and richest (6–10).

### 2.3. Statistical approach

Our statistical approach begun with a Principal Components Analysis (PCA) to reduce the dimensionalities of the 12 items measuring client satisfaction. We then conducted a descriptive analysis of the outcomes and covariates. Last, we estimated univariate and bivariate probit regression models to assess client dissatisfaction influence on contraceptive discontinuation, adjusted for service delivery point context and individual characteristics.

#### 2.3.1. Principal component analysis of service quality

PCA is a data reduction technique used to summarize multivariate correlated data into fewer dimensions (latent variables) that capture the maximum possible information from the original variables [25]. All twelve potential service problems were included in the PCA as binary variables. The scree plot and eigenvalues above one informed the number of necessary components. The PCA identified four components representing clients’ dissatisfaction with family planning services; see Fig A-2 in S1 Appendix and Table A-2 in S1 Appendix. Item loadings above 0.4 were included in each component [26]. An orthogonal varimax rotation was applied to optimize understanding of the correlated data structure. The loadings of each item per component are given in Table 1. The first component, availability, represents the range of services available and the availability of medicines and contraceptives. The second component represents the client’s privacy while receiving family planning services. The third component, convenience of family planning services, captures the hours and days of service and the length of time clients have to wait to see a provider. Finally, the fourth component captures the client’s perception of the cleanliness of the facility and staff treatment.

**Table 1:** Principal component analysis (PCA), component loadings, orthogonal varimax rotation of loading matrix after PCA.

	Orthogonal Varimax Rotation				
Item	Comp. 1 =	Comp. 2 =	Comp. 3 =	Comp. 4 =	Unexplained
	Availability	Privacy	Convenience	Clean/Staff treatment	
Range of services available	**0.513**	0.102	-0.003	-0.110	0.429
Availability of medicines	**0.557**	0.007	-0.026	-0.065	0.408
Availability of contraceptives	**0.475**	-0.065	0.093	0.023	0.518
Visual privacy during examination	0.003	**0.683**	-0.013	0.113	0.193
Auditory privacy during examination	0.015	**0.688**	0.001	-0.060	0.215
Hours of Service	0.018	0.030	**0.586**	-0.106	0.493
Days of service	0.178	0.066	**0.438**	0.060	0.539
Time waited to see a provider	-0.046	-0.136	**0.515**	0.235	0.559
Cleanliness of the facility	-0.214	0.113	0.134	**0.645**	0.407
Staff treatment	0.163	-0.063	-0.222	**0.534**	0.539
Amount of explanation received	0.234	-0.050	0.148	0.307	0.676
Cost of services or treatment	0.198	-0.051	-0.318	0.319	0.704

Note: Bold values indicate the item is loading on the component. Obs. = 775.

With the four components identified, composite measures of clients’ dissatisfaction with family planning services were generated for each in order to have a summary metric per component. First, we added the items with eigenvalues above the 0.4 threshold for each component. Then if the client’s composite measure was positive, i.e., greater than zero, it was defined as a problem; otherwise, it was coded zero, indicating no problem was encountered.

#### 2.3.2. Descriptive analysis

We present the percentage of clients who discontinued (or continued) contraception between baseline and follow-up and the percentage of clients who experienced problems with availability, privacy, convenience, or cleanliness/staff treatment during their visit (Table 2). Next, we tabulate the distribution of individual and service delivery point characteristics and how these co-vary with discontinuation and clients’ experience with the four types of problems (Table 2 and Table A-3 in S1 Appendix). We perform bivariate chi-squared tests of differences in these characteristics for the outcomes of interest. Differences were computed between discontinuers and continuers, and between women experiencing problems with any of the composite measures capturing the quality of family planning services compared to those with no problems.

**Table 2:** Sample characteristics of family planning clients stratified by contraceptive discontinuation status.

Variables	Total		Contraceptive discontinuation		
			Discontinuer	Continuer	*χ*^2^ test
	Obs.	%	%	%	*χ*^2^-statistic (p-value)
Contraceptive discontinuation					
Discontinuer	145	18.2			
Continuer	652	81.8			
Reports problems with					
Availability	797	11.9	13.1	11.7	0.24 (0.627)
Privacy	797	3.5	2.8	3.7	0.30 (0.585)
Convenience	797	13.6	13.1	13.7	0.03 (0.862)
Clean/Staff treatment	797	5.8	9.7	4.9	**4.91 (0.027)** **
Managing authority					
Private	123	15.9	19.9	15.0	2.01 (0.157)
Public	650	84.1	80.1	85.0	
Contraceptive stocks					
In-stock	672	86.9	85.8	87.2	0.19 (0.663)
Not offered/Out-of-stock	101	13.1	14.2	12.8	
Fee for FP methods					
No	449	58.1	48.9	60.1	**5.93 (0.015)** **
Yes	324	41.9	51.1	39.9	
Provider discussed FP					
No	175	22.0	29.0	20.4	**5.08 (0.024)** **
Yes	622	78.0	71.0	79.6	
Used the baseline method before the visit					
No	221	27.7	29.0	27.5	0.14 (0.713)
Yes	576	72.3	71.0	72.5	
Baseline method					
Long-acting reversible contraception	248	31.1	42.1	28.7	**9.92 (0.002)** **
Short-acting reversible contraception	549	68.9	57.9	71.3	
Heard FP in radio or TV (last 3 months)					
No	233	29.3	31.0	28.9	0.26 (0.614)
Yes	562	70.7	69.0	71.1	
CHW talked about FP recently					
No	692	87.0	86.2	87.2	0.11 (0.740)
Yes	103	13.0	13.8	12.8	
Marital status					
In union	720	90.3	88.3	90.8	0.86 (0.353)
Not in union	77	9.7	11.7	9.2	
Age					
18–24	203	25.5	28.3	24.8	**8.30 (0.016)** **
25–34	399	50.1	56.6	48.6	
35–49	195	24.5	15.2	26.5	
Parity					
None-1	184	23.1	35.2	20.4	**14.71 (0.001)** ***
2–3	387	48.6	42.1	50.0	
4+	226	28.4	22.8	29.6	
Education					
None/primary	266	33.4	40.0	31.9	3.91 (0.142)
Post-primary/Secondary	370	46.4	40.0	47.9	
College/University	161	20.2	20.0	20.2	
Wealth					
Poorest	298	37.6	43.4	36.3	2.81 (0.245)
Middle	395	49.8	46.2	50.6	
Richest	100	12.6	10.3	13.1	
Discussed FP w/ partner (last 6 months)					
No	268	33.7	42.4	31.7	**5.95 (0.015)** **
Yes	528	66.3	57.6	68.3	** **

Notes: Bold values indicate significant differences between discontinuers and continuers. Table A-8 in S1 Appendix provides a detailed description of these variables and whether they were retrieved from the CEI or SDP questionnaire.

*** p<0.01

** p<0.05

* p<0.1.

#### 2.3.3. Probit regression models

We constructed a univariate probit model (Table 3) and a bivariate probit model (Table 4) to identify whether contraceptive discontinuation was related to clients’ dissatisfaction with family planning services received. In the univariate probit, presented in Eq 1, the outcome of interest $y_{1}=1(y_{1i}^{*}>0)$ is a binary indicator for whether client *i* discontinued the use of contraception—it equaled zero if the client continued using the same method of contraception. We regress this outcome on a set of service delivery point (*SDP*) and individual characteristics (*CEI*), controlling for unobservable characteristics at the country level by adding country dummies (*ξ*_*s*_). The parameter of interest in this equation is *γ*_1_, which measures the difference in the probability of discontinuing contraception between clients who were dissatisfied with the family planning services they received at the facility and satisfied clients. A set of four indicator variables captured this dissatisfaction, $y_{2}=1(y_{2i}^{*}>0)$, for reporting a problem with: i) availability, or ii) privacy, or iii) convenience, or iv) cleanliness and/or staff treatment. We add each dissatisfaction variable one at a time and also the four together at the same time.

**Table 3:** Results from probit regression models of client dissatisfaction with family planning services on contraceptive discontinuation.

		Outcome: Contraceptive Discontinuation			
		Reports problem w/ availability (*ref*. *No*)	Reports problem w/ privacy *(ref*. *No)*	Reports problem w/ convenience *(ref*. *No)*	Reports problem w/ clean/staff treatment *(ref*. *No)*
Model 1	Coeff.	0.137 [-0.228–0.502]	-0.372 [-0.982–0.239]	0.003 [-0.342–0.348]	0.341* [-0.056–0.737]
	Avg. Marg. Effects	0.033 [-0.055–0.121]	-0.090 [-0.237–0.057]	0.001 [-0.083–0.084]	0.082* [-0.013–0.178]
Model 2	Coeff.	0.134 [-0.212–0.479]			
	Avg. Marg. Effects	0.032 [-0.051–0.116]			
Model 3	Coeff.		-0.266 [-0.855–0.323]		
	Avg. Marg. Effects		-0.065 [-0.207–0.078]		
Model 4	Coeff.			0.031 [-0.297–0.359]	
	Avg. Marg. Effects			0.007 [-0.072–0.087]	
Model 5	Coeff.				0.320 [-0.071–0.711]
	Avg. Marg. Effects				0.078 [-0.017–0.172]

Notes: Reporting probit coefficients (Coeff.) with 95% CIs computed with robust standard errors in brackets. Reporting average marginal effects (Avg. Marg. Effects) with CIs computed with the delta-method in brackets. All models control for country fixed effects. Control variables included were: managing authority, contraceptive stocks, fee for family planning methods, provider discussed family planning, used the baseline method before the visit, baseline method, heard about family planning in the radio or TV, talked recently with a CHW about family planning, marital status, age, parity, education, household wealth, and discussed about family planning with partner. Full model is presented in Table A-4 in S1 Appendix.

*** p<0.01

** p<0.05

* p<0.1.

**Table 4:** Regression results from the seemingly unrelated bivariate probit models.

	Model 2		Model 3		Model 4		Model 5	
Variables	Disconti-nuer	Problem w/ availability	Disconti-nuer	Problem w/ privacy	Disconti-nuer	Problem w/ convenience	Disconti-nuer	Problem w/ clean/staff treatment
Inverse hyperbolic tangent of $\hat{\rho }$	0.111		-0.164		0.031		0.152	
	[-0.070–0.292]		[-0.406–0.078]		[-0.146–0.208]		[-0.044–0.348]	
**Managing authority (*ref*. *Private*)**								
Public	-0.151	**0.618** **	-0.158	0.082	-0.155	**0.819** ***	-0.157	0.024
	[-0.493–0.190]	[0.122–1.114]	[-0.500–0.185]	[-0.547–0.711]	[-0.497–0.186]	[0.276–1.362]	[-0.501–0.187]	[-0.468–0.517]
**Contraceptive stocks (*ref*. *In-stock*)**								
Not offered/Out-of-stock	0.130	**0.665** ***	0.130	-0.056	0.128	-0.231	0.126	-0.254
	[-0.197–0.457]	[0.321–1.009]	[-0.195–0.456]	[-0.565–0.452]	[-0.198–0.455]	[-0.642–0.181]	[-0.201–0.453]	[-0.803–0.296]
**Fee for FP methods (*ref*. *No*)**								
Yes	0.149	-0.066	0.145	0.245	0.147	-0.079	0.145	-0.066
	[-0.150–0.448]	[-0.445–0.313]	[-0.152–0.443]	[-0.219–0.709]	[-0.151–0.446]	[-0.413–0.255]	[-0.154–0.443]	[-0.451–0.318]
**Provider discussed FP (*ref*. *No*)**								
Yes	**-0.298** **	0.158	**-0.297** **	0.078	**-0.301** **	-0.080	**-0.299** **	-0.153
	[-0.558 - -0.037]	[-0.149–0.466]	[-0.557 - -0.037]	[-0.342–0.497]	[-0.561 - -0.041]	[-0.373–0.212]	[-0.560 - -0.039]	[-0.505–0.200]
**Used the baseline method before the visit (*ref*. *No*)**								
Yes	0.112	0.029	0.112	0.048	0.111	**-0.338** **	0.110	0.113
	[-0.155–0.379]	[-0.263–0.320]	[-0.155–0.379]	[-0.362–0.458]	[-0.155–0.378]	[-0.608 - -0.067]	[-0.157–0.376]	[-0.255–0.480]
**Baseline method (*ref*. *LARC*)**								
Short-acting reversible contraception	**-0.410** ***	-0.061	**-0.411** ***	**-0.569** ***	**-0.409** ***	-0.112	**-0.406** ***	-0.171
	[-0.653 - -0.166]	[-0.343–0.220]	[-0.655 - -0.167]	[-0.958 - -0.180]	[-0.653 - -0.165]	[-0.379–0.155]	[-0.650 - -0.162]	[-0.495–0.153]
**Heard FP in radio or TV (last 3 months) (*ref*. *No*)**								
Yes	-0.030	-0.027	-0.029	0.054	-0.030	-0.039	-0.034	**-0.263** *
	[-0.274–0.213]	[-0.311–0.256]	[-0.272–0.214]	[-0.379–0.488]	[-0.273–0.213]	[-0.319–0.241]	[-0.276–0.209]	[-0.575–0.050]
**CHW talked about FP recently (*ref*. *No*)**								
Yes	0.039	0.100	0.034	0.162	0.038	**-0.517** **	0.038	0.050
	[-0.288–0.365]	[-0.268–0.467]	[-0.293–0.361]	[-0.332–0.656]	[-0.289–0.364]	[-0.918 - -0.115]	[-0.287–0.364]	[-0.391–0.492]
**Marital status (*ref*. *In union*)**								
Not in union	0.002	-0.002	0.005	0.309	0.005	-0.008	0.005	0.152
	[-0.387–0.392]	[-0.407–0.403]	[-0.383–0.393]	[-0.195–0.814]	[-0.384–0.394]	[-0.405–0.389]	[-0.386–0.395]	[-0.355–0.658]
**Age (*ref*. *18–24*)**								
25–34	**0.284** *	**0.320** *	**0.282** *	**0.596** ***	**0.283** *	0.254	**0.285** *	0.166
	[-0.009–0.576]	[-0.005–0.645]	[-0.008–0.573]	[0.188–1.005]	[-0.008–0.575]	[-0.064–0.572]	[-0.008–0.577]	[-0.211–0.542]
35–49	-0.101	**0.347** *	-0.101	**0.499** *	-0.101	**0.594** ***	-0.098	0.375
	[-0.498–0.297]	[-0.056–0.751]	[-0.495–0.294]	[-0.060–1.058]	[-0.497–0.295]	[0.196–0.992]	[-0.494–0.299]	[-0.074–0.824]
**Parity (*ref*. *None-1*)**								
2–3	**-0.535** ***	0.089	**-0.528** ***	-0.059	**-0.533** ***	-0.088	**-0.534** ***	0.013
	[-0.834 - -0.236]	[-0.255–0.433]	[-0.826 - -0.231]	[-0.443–0.326]	[-0.831 - -0.234]	[-0.413–0.238]	[-0.833 - -0.234]	[-0.358–0.383]
4+	**-0.580** ***	-0.158	**-0.581** ***	**-0.912** ***	**-0.581** ***	-0.147	**-0.581** ***	**-0.499** **
	[-0.974 - -0.186]	[-0.584–0.268]	[-0.973 - -0.188]	[-1.520 - -0.303]	[-0.974 - -0.188]	[-0.566–0.272]	[-0.976 - -0.186]	[-0.958 - -0.040]
**Education (*ref*. *None/primary*)**								
Post-Primary/Secondary	**-0.343** ***	**0.277** *	**-0.351** ***	-0.003	**-0.345** ***	0.112	**-0.346** ***	-0.255
	[-0.596 - -0.089]	[-0.014–0.568]	[-0.605 - -0.098]	[-0.444–0.438]	[-0.599 - -0.091]	[-0.152–0.376]	[-0.600 - -0.091]	[-0.593–0.082]
College/University	**-0.383** **	-0.064	**-0.389** **	-0.014	**-0.382** **	-0.025	**-0.380** **	0.066
	[-0.723 - -0.043]	[-0.487–0.359]	[-0.727 - -0.050]	[-0.604–0.576]	[-0.722 - -0.043]	[-0.420–0.370]	[-0.720 - -0.041]	[-0.360–0.493]
**Wealth (*ref*. *Poorest (1–3)*)**								
Middle (4–5)	-0.127	0.038	-0.121	0.018	-0.124	-0.194	-0.123	-0.055
	[-0.372–0.119]	[-0.252–0.329]	[-0.367–0.125]	[-0.387–0.423]	[-0.370–0.122]	[-0.465–0.076]	[-0.369–0.123]	[-0.385–0.275]
Richest (6–10)	-0.211	0.007	-0.213	-0.164	-0.215	-0.199	-0.217	-0.131
	[-0.595–0.173]	[-0.438–0.451]	[-0.597–0.172]	[-0.821–0.493]	[-0.600–0.170]	[-0.614–0.216]	[-0.603–0.168]	[-0.633–0.372]
**Discussed FP w/ partner (last 6 months) (*ref*. *No*)**								
Yes	-0.250*	**-0.300** **	-0.247*	-0.191	-0.247*	-0.049	-0.245*	-0.200
	[-0.503–0.003]	[-0.557 - -0.042]	[-0.500–0.006]	[-0.612–0.229]	[-0.500–0.006]	[-0.311–0.213]	[-0.497–0.008]	[-0.525–0.125]
Constant	0.175	**-1.800** ***	0.174	**-1.637** ***	0.176	**-1.087** ***	0.175	**-1.248** ***
	[-0.392–0.743]	[-2.550 - -1.050]	[-0.394–0.741]	[-2.589 - -0.685]	[-0.392–0.744]	[-1.857 - -0.316]	[-0.394–0.745]	[-2.060 - -0.435]
Observations	765	765	765	765	765	765	765	765

Notes: Reporting probit coefficients with 95% Cis computed with robust standard errors in brackets. All models control for country fixed effects. $\hat{\rho }$ is the inverse hyperbolic tangent of the correlation between the error terms shown in Eq 2.

*** p<0.01

** p<0.05

* p<0.1.

Table 4 presents the results of the seemingly unrelated bivariate probit regression model of discontinuing contraception and experiencing a problem with availability, privacy, convenience, and cleanliness and/or staff treatment. If $\hat{\rho }$ is not statistically significant, it means the estimation for these two outcomes is better suited with a univariate probit model than with a bivariate estimation. The Wald test of $\hat{\rho }$ shows that the decision to discontinue contraception is not significantly interrelated with any of the measures of client dissatisfaction with family planning services. Hence, this means that the univariate probit model fits better than the seemingly unrelated bivariate probit model.


$$y_{1i}=\alpha_{1}+\gamma_{1}y_{2i}+\lambda_{1}{SDP}_{1i}+\theta_{1}{CEI}_{1i}+\xi_{s}+\mu_{1}$$ [Eq 1]

We hypothesize that contraceptive discontinuation and clients’ dissatisfaction are not exogenously related. To test this hypothesis, we estimated a seemingly unrelated bivariate probit model, in which we characterized the interrelationship between contraceptive discontinuation and client dissatisfaction with family planning services as follows:


$$\begin{array}{c} y_{1i}=\alpha_{1}+\lambda_{1}{SDP}_{1i}+\theta_{1}{CEI}_{1i}+\xi_{s}+\mu_{1}\\ y_{2i}=\alpha_{2}+\lambda_{2}{SDP}_{2i}+\theta_{2}{CEI}_{2i}+\xi_{s}+\mu_{2} \end{array}$$ [Eq 2]

Similar to Eq 1, both equations of the seemingly unrelated bivariate probit were a function of the same vectors *SDP* and *CEI*, and of country fixed effects, *ξ*_*s*_. In the seemingly unrelated bivariate probit model, we impose no structural shifts between $y_{1i}^{*}$ and $y_{2i}^{*}$ beyond the error terms, *μ*_1_ and *μ*_2_. The seemingly unrelated bivariate probit model performs a joint estimation of the two binary dependent variables with the error terms jointly distributed as standard bivariate normal variables and their correlation coefficient being $\hat{\rho }$. If $\hat{\rho }$ is statistically significant, then the unobservable characteristics that influence contraceptive discontinuation are significantly correlated with the unobservable characteristics that influence client dissatisfaction with family planning services, which signals that a seemingly unrelated bivariate probit model is better than a univariate probit model.

As a robustness check, we estimated probit regression models where the outcome of interest, discontinuation, was regressed on each of the twelve client dissatisfaction items both independently and jointly (Table A-5 in S1 Appendix).

## 3. Results

The percentage distributions of clients who discontinued contraception between baseline and follow-up and who experienced a problem with family planning services at baseline are presented in Table 2. Across 797 clients interviewed in urban health facilities in Kenya, Nigeria, and Burkina Faso, 18.2% of clients stopped using contraception four to six months after they were prescribed a modern contraceptive. Convenience, which captures problems with the hours and days of service and time waited to see a provider, was the most common problem experienced across clients at 13.6%. The second most common problem was experiencing an issue with the availability of at least one of the following items: services, medicines, or contraceptives, reported by 11.9% of clients. Cleanliness of the facility or staff treatment was a problem for 5.8% of clients. Finally, 3.5% of clients reported a problem with auditory or visual privacy during their family planning visit. These estimates are presented at the country level in Table A-6 in in S1 Appendix.

Table 2 also summarizes the characteristics of the service delivery points where clients obtained family planning services and their own characteristics, both measured at baseline. Most clients attended a public health facility (84.1%). Out of all clients included in the analysis, 13.1% reported that their facility was out-of-stock of contraceptives at the time of visit. Two-fifths (41.9%) of the clients had to pay a fee to obtain family planning services; and more than three-quarters (78.0%) indicated their provider discussed family planning during their visit. Note that even though their facility visit reason was for family planning services, they could have obtained supplies from a pharmacist who may not have provided specific information about contraceptive methods. Almost three-quarters of clients had used the baseline method before their visit (72.3%) and most reported a short-acting method at baseline (68.9%). The majority of clients (70.7%) reported they had heard about family planning on either television or radio in the past three months. Because these are urban clients and community health workers tend to be rural, only a small share (13.0%) reported being visited by a community health worker who discussed family planning with them. Almost all clients in the sample (90.3%) were married; and two-thirds (66.3%) had discussed family planning with their partner in the last six months. On average, women were 29.8 years of age, had 2.8 children, and ranked their household well-being at the fourth out of a ten-step ladder. One-third of clients had no or primary education (33.4%), 46.4% had post-primary or secondary education, and the remaining 20.2% had either college or university education.

Bivariate chi-squared tests of difference in service delivery point and individual characteristics between contraceptive discontinuers and continuers are also in Table 2. We find that the percentage of clients who experienced a problem with the cleanliness and/ or staff treatment among discontinuers was higher than among continuers—9.7% and 4.9%, respectively. We also find significant differences when asked about having to pay a fee for family planning services (p<0.05), discussing family planning with the provider (p<0.05), baseline method (p<0.05), across age categories (p<0.05), parity levels (p<0.01), and between clients that discussed family planning with their partners and those that did not (p<0.05). Table A-3 in S1 Appendix shows similar computations as Table 2, except clients are stratified according to their dissatisfaction with family planning services.

Next, we present the univariate probit regression model results of the influence of client dissatisfaction with family planning services—proxied by our four measures: availability, privacy, convenience, and facility cleanliness/staff treatment—on contraceptive discontinuation (Table 3). Clients who experienced a problem with the availability, privacy, or convenience during their family planning visit did not have a significantly different probability of discontinuation than clients who did not experience such problems (Models 1 to 4). We find that the adjusted probability of discontinuation for clients who reported a problem with the cleanliness of the facility and/ or staff treatment was 8.2 percentage-points higher than for clients who did not report such a problem. We focused on Model 1 (instead of Model 5) because this specification reported the lowest Akaike information criterion (AIC), suggesting Model 1 fitted the data best than Models 2–5.

## 4. Discussion

The consistent and correct use of family planning methods can reduce unplanned pregnancies and unwanted births. Circumstances in facility environment and provider care can play a role in motivating continuous use of contraception. This study investigated the relationship between client dissatisfaction with family planning services and contraceptive discontinuation among female clients who attended a health facility seeking family planning services in urban sites in Kenya, Nigeria, and Burkina Faso. We specified a set of probit and seemingly unrelated bivariate probit models to test our hypothesis that the relationship between these two variables is not endogenous. We find that clients reporting facility cleanliness and/or staff treatment as problematic influenced their continued use four to six months later. Clients’ impressions of the facility and their providers likely informed their service expectations and confidence in their contraceptive methods. The average difference in the probability of discontinuing contraception was 8.2 percentage-points between dissatisfied women and satisfied women. Our findings are not that far from other studies that investigated this relationship, even though these are few. For example, RamaRao et al. (2003) found that women who received high-quality family planning care had a twelve percentage-point higher probability of continuing using contraception than women who received low-quality care.

The literature measuring the effects of client dissatisfaction with family planning services (or quality of family planning care) on the use of contraception in the context of developing countries is limited. Most of this research relies on population surveys, and these surveys rarely collect information about the healthcare system environment. As a result, it is difficult to identify the healthcare facility where women obtained their family planning services unless the study relies on client exit interviews, like PMA Agile. Jain et al. (2019) found that clients who received high-quality family planning services reported higher odds of continued contraceptive use than those who received low quality. In addition, measuring the quality of care of family planning services is difficult, and there is no consensus over one standardized metric. As a result, the evidence of the effects of family planning quality of care on contraceptive discontinuation is mixed. Some studies found an association between quality and discontinuation [27–29], while others found no significant effect [14, 30].

Significant individual factors associated with contraceptive discontinuation were parity and education. Women of higher parity were less likely to discontinue contraception than women with no more than one child. A similar association was found with education; women with post-primary/secondary or college/university degrees were less likely to discontinue contraception than women with none/primary education. Perhaps women with more children and more education were more motivated to use and to continue using contraception for personal and lifestyle reasons, but also because they may have had all the children they want. These findings are aligned with prior literature where education and parity were also significant factors of contraceptive discontinuation [9, 12]. By including service delivery point characteristics, we are able to detect that client who discussed family planning services with their provider had a significantly lower predicted probability of discontinuing contraception than clients who did not hold such discussions.

Our study offers several strengths. First, all women included in our analysis attended the health facility seeking family planning services and the majority were prescribed or received a modern contraceptive at the baseline interview, which allowed us to link their service quality perceptions to the facility at the time those services were provided. The facility characteristics included in our analysis correspond to those key proxies of adequate service provision by public and private providers—contraceptive supplies in stock, fee charges, and provider counseling. The problems that women ranked were captured at the time she completed her family planning visit and exited the facility; thus, they are not subjective to significant recall bias. Second, contraceptive discontinuation is measured prospectively by following up with the same client cohorts four to six months after the initial visit. The use of prospective data ensures our discontinuation measure is not affected by misreporting and, to a lesser extent, by selection bias [22]. Women were asked about their current contraceptive behavior in both survey rounds. This offers advantages of reporting accuracy as compared to the contraceptive calendar, where only women who are eligible for the survey sample provide their use histories. However, selection bias could be introduced through phone ownership; women needed to provide a phone number to receive a call back for the follow-up interview. Based on all female clients consenting to follow-up, the percent owning phones or providing valid access numbers was above 60% in the three countries and follow-up rates ranged from 61.9% in Nigeria to 88.9% in Kenya. Third, our data measure socioeconomic factors present at a time prior to the discontinuation episode. Fourth, in our model specification, the unobserved characteristics that influence the decision to discontinue contraception and the unobservable characteristics that influence experiencing a problem with family planning services were allowed to be correlated, a closer approximation to the real world where individual decisions are not taken in siloes.

Our study has its limitations, one being its generalizability: limiting our sample to women visiting the facility for family planning reasons reduced our sample size and required combining data from Kenya, Nigeria, and Burkina Faso sites. However, we included country-fixed effects to control for country-level factors that are unobservable but may influence our outcomes of interest. We also checked that the composition of contraceptive methods used at baseline was similar across countries, as well as the percentage of discontinuation episodes. A second limitation was that we could not capture the number of previous family planning visits a woman had prior to the survey. We cannot document her satisfaction with prior services. Her prior experiences could also inform her contraceptive plans and behavior; we assumed that the most recent interaction carries a higher weight with her subsequent contraceptive behavior.

A third limitation related to the prior use of contraception is that we cannot account for the influence of the duration of use of the contraceptive method reported at baseline on a woman’s decision to continue or stop using contraception. However, the PMA Agile questionnaire asked women who were prescribed or dispensed contraception during the baseline visit to indicate whether the method was new or not (657 clients). The remaining 140 clients that were not prescribed or dispensed contraception on the day of the interview were asked to indicate the method they were using at that time (140 clients). We used this information to construct a variable measuring whether the client had used the baseline method before the visit in order to control for prior use of contraception. In addition, we computed discontinuation rates by baseline method (Table A-7 in S1 Appendix).

A fourth limitation is we do not know why women stopped using contraception because the reason for discontinuation was not asked in the follow-up survey. It is possible some women stopped using contraception because they wanted to have a child. However, we do know that only 3.4% of the women in the sample were nulliparous, 19.7% had at least one child, and the interviews were conducted only four to six months apart. A fifth limitation is the use of the PCA to identify a latent structure that can categorize the dissatisfaction with family planning services items into fewer dimensions based on the correlation between these items. This method is empirically driven and does not always provide an obvious interpretation of the combination of variables making up a component.

A fifth limitation is the potential of self-reported response bias. Women were asked to report their satisfaction right after their family planning visit and at the facility where the visit occurred. This approach could have introduced social desirability bias, meaning women felt obliged to provide positive answers because they were surveyed at the same facility. Women could have also responded systematically more positively or negatively to all client dissatisfaction questions, which could also have triggered skewed response distributions. Unfortunately, we did not have enough clients per facility to construct an alternate measure of client dissatisfaction at the facility level to control for self-reported response bias.

A final limitation is that switchers were dropped from our analytical sample. However, before dropping them, we measured their levels of dissatisfaction with family planning services. We noticed their pattern was different than the pattern from continuers and stoppers. We were not comfortable grouping switchers with stoppers or switchers with continuers. As a sensitivity analysis, we estimated a multinomial probit regression model. We found that the relative decision to stop (relative to continue) increased for clients dissatisfied with clean and or staff treatment (relative to satisfied clients), consistent with the findings from the analytical sample we used in this study.

Previous research on this topic has primarily focused on identifying individual factors of contraceptive discontinuation and on measuring discontinuation rates within a specific timeframe (e.g., Moreau, Bouyer [31], Ali, Cleland [3], Barden-O’Fallon, Speizer [13], Moreau, Cleland [18]). Few studies have focused on measuring the association of the quality of care of family planning services on contraceptive discontinuation, mainly due to data limitations and lack of consensus on how to measure quality (e.g., Feeser, Chakraborty [16], Chakraborty, Chang [32], Jain, Aruldas [27]). Our study adds the client’s perspective, as a dimension of quality of care, to the contraceptive discontinuation literature and includes characteristics of the health facility where we know each client obtained their contraception. Even though our sample is not generalizable to the female population seeking family planning services at a large, our pooled sample size can be taken as representative of urban clients using family planning services in Kenya, Nigeria, and Burkina Faso. The baseline method composition across contraceptive users included in our analytic sample is similar for the three countries, which suggests reasonably homogenous contraceptive behavior. In general, we find that client satisfaction with family planning services can influence women’s decision to continue using contraception.

## 5. Conclusions

Experiencing problems with the cleanliness of the facility and (or) staff treatment during a family planning visit significantly influenced clients’ future decisions to discontinue modern contraception. The average difference in the probability of discontinuing contraception was 8.2 percentage-points between dissatisfied women and satisfied women. Experiencing a problem with the availability, privacy, or convenience during a family planning visit was not significantly associated with the decision to discontinue the use of contraception. We were puzzled to find that these three measures of client dissatisfaction with family planning services, which we defined as proxies of family planning quality of care, were not significantly associated with contraceptive discontinuation. This finding suggests that we need further research to better measure the quality of care and how women interpret quality when receiving family planning services.

## Supporting information

S1 Appendix (DOCX)
