# Supplementary material for: The relationship between client dissatisfaction and contraceptive discontinuation among urban family planning clients in three sub-Saharan African countries
Source: PLoS One. 2022 Aug 22;17(8):e0271911. doi: 10.1371/journal.pone.0271911 (PMC9394817; doi:10.1371/journal.pone.0271911)
Supplement: S1 Appendix — (DOCX) [file pone.0271911.s001.docx]

Appendix

Tables

[Table A- 1: Data collection dates by country and client cohort 2](#_Toc107324877)

[Table A- 2: Component Eigenvalues after PCA 5](#_Toc107324878)

[Table A- 3: Percent distribution of urban client sample characteristics stratified by experience with service problems 6](#_Toc107324879)

[Table A- 4: Results from probit regression models of client dissatisfaction with family planning services on contraceptive discontinuation 8](#_Toc107324880)

[Table A- 5: Regression results from the univariate probit 11](#_Toc107324881)

[Table A- 6: Percentage of clients who discontinued contraception and experienced problems during a family planning visit by country 14](#_Toc107324882)

[Table A- 7: Percentage of clients who discontinued contraception by method type used in the baseline interview 15](#_Toc107324883)

[Table A- 8: List of variables used in the analysis 16](#_Toc107324884)

Figures

[Figure A- 1: Composition of contraceptive methods used by urban family planning client sample 3](#_Toc107324885)

[Figure A- 2: Scree plot for eigenvalues after principal component analysis 4](#_Toc107324886)

# Table A- 1: Data collection dates by country and client cohort

|  | Round 1 cohort | | Round 2 cohort | |
| --- | --- | --- | --- | --- |
| Location | Baseline (in-person) | Follow-up (phone) | Baseline (in-person) | Follow-up (phone) |
| Kenya | Mar.-Aug. 2018 | Sep.-Dec. 2018 | Feb.-Jun. 2019 | Jul.-Oct. 2019 |
| Nigeria | Mar.-Aug. 2018 | Sep.-Nov. 2018 | Feb.-May. 2019 | Jun.-Aug. 2019 |
| Burkina Faso | Aug.-Oct. 2018 | Feb.-Apr. 2019 | Jun.-Sep. 2019 | Oct.-Nov. 2019 |
|  |  |  |  |  |

# Figure A- 1: Composition of contraceptive methods used by urban family planning client sample


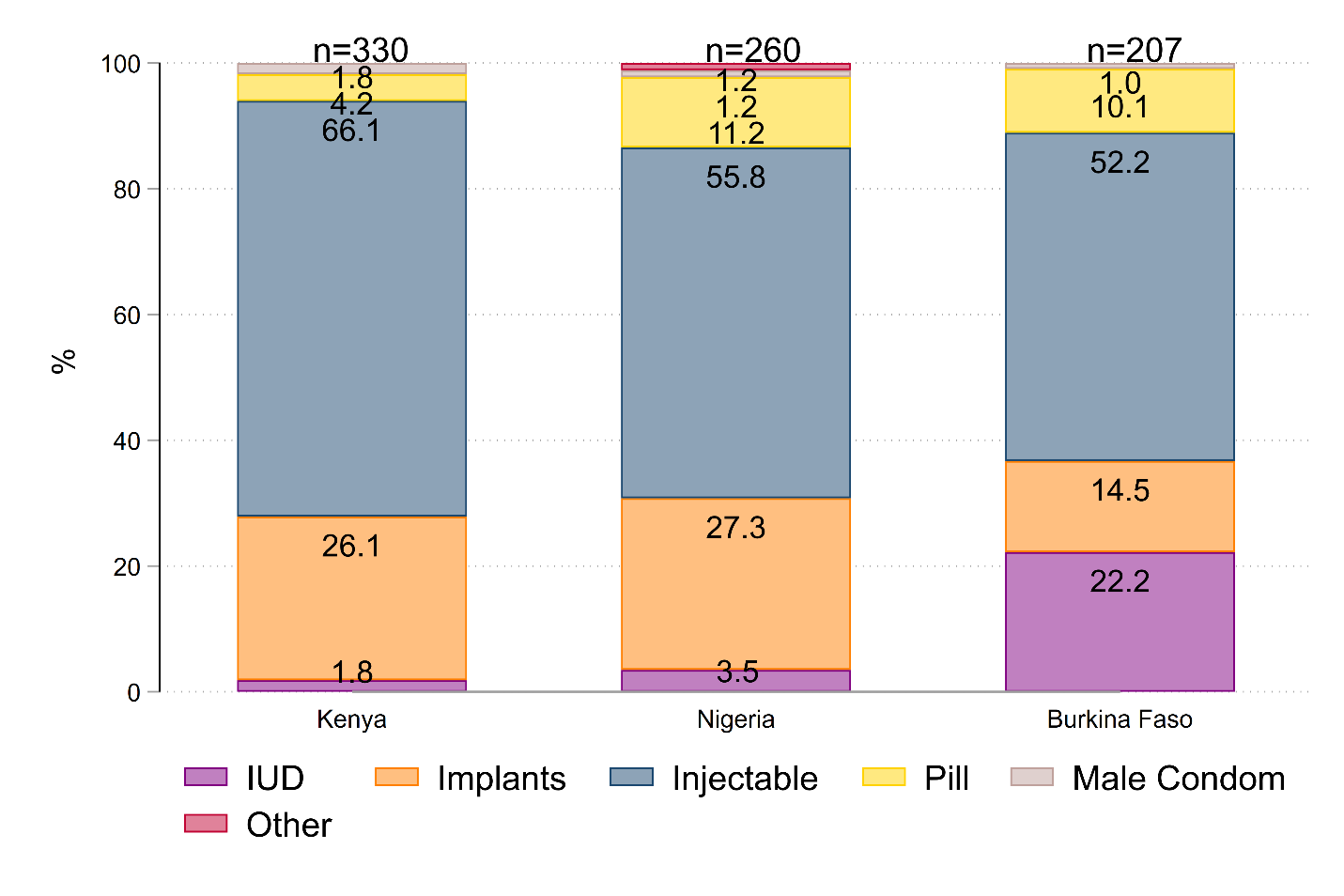


# Figure A- 2: Scree plot for eigenvalues after principal component analysis


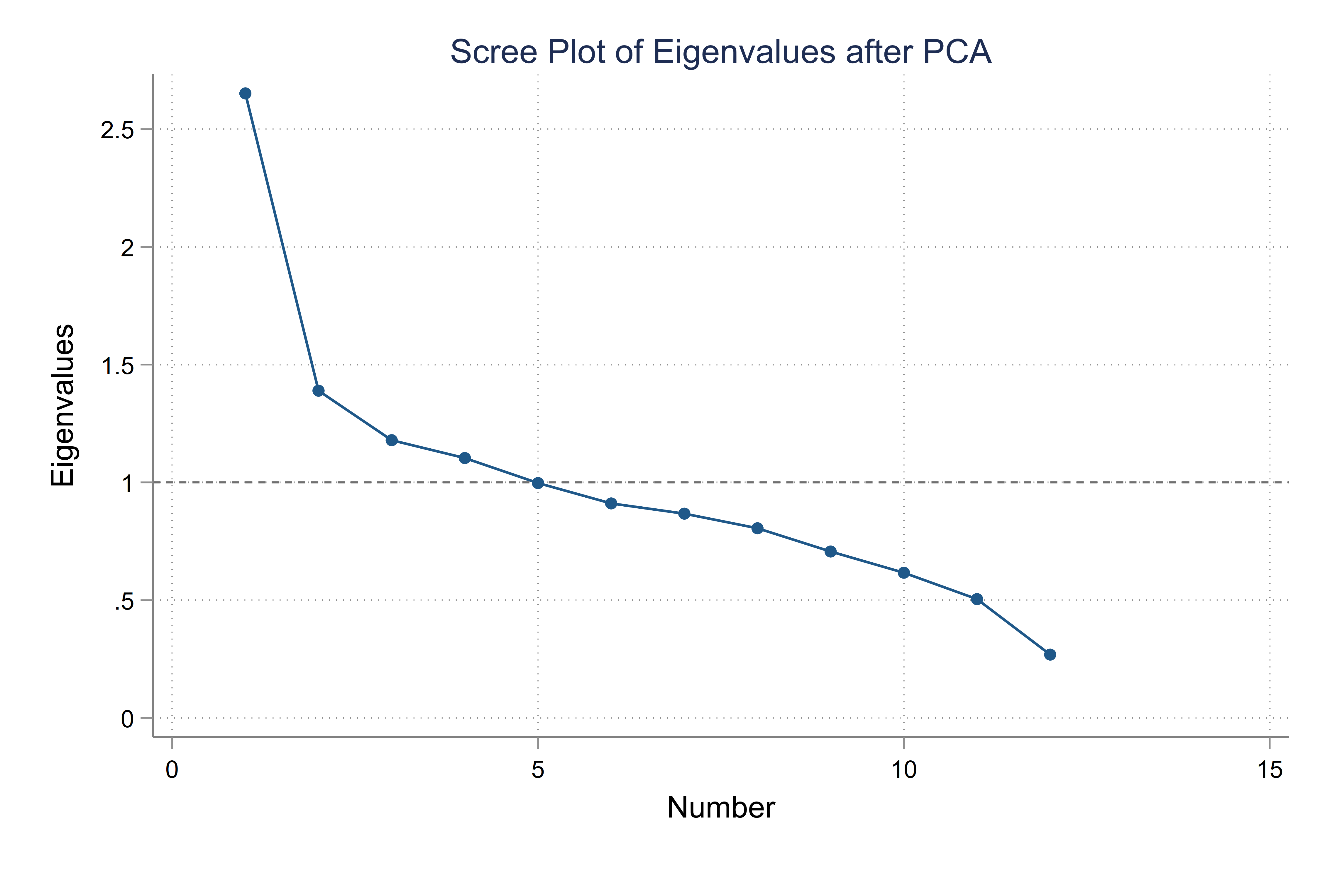


# Table A- 2: Component Eigenvalues after PCA

| **Component** | **Eigenvalue** | **Difference** | **Proportion** | **Cumulative** |
| --- | --- | --- | --- | --- |
| Comp1 | 2.652 | 1.262 | 0.221 | 0.221 |
| Comp2 | 1.389 | 0.210 | 0.116 | 0.337 |
| Comp3 | 1.179 | 0.076 | 0.098 | 0.435 |
| Comp4 | 1.103 | 0.106 | 0.092 | 0.527 |
| Comp5 | 0.997 | 0.086 | 0.083 | 0.610 |
| Comp6 | 0.911 | 0.044 | 0.076 | 0.686 |
| Comp7 | 0.867 | 0.062 | 0.072 | 0.758 |
| Comp8 | 0.805 | 0.099 | 0.067 | 0.825 |
| Comp9 | 0.706 | 0.090 | 0.059 | 0.884 |
| Comp10 | 0.616 | 0.112 | 0.051 | 0.936 |
| Comp11 | 0.504 | 0.236 | 0.042 | 0.978 |
| Comp12 | 0.269 | . | 0.022 | 1.000 |
|  |  |  |  |  |
| Observations | 775 |  |  |  |
|  |  |  |  |  |

# Table A- 3: Percent distribution of urban client sample characteristics stratified by experience with service problems

| Variables | Client reports experience problem with | | | | | | | | | | | |
| --- | --- | --- | --- | --- | --- | --- | --- | --- | --- | --- | --- | --- |
|  | Availability | | | Privacy | | | Convenience | | | Clean/Staff treatment | | |
|  | Problem | No Problem | $\chi^{2}$-test | Problem | No Problem | $\chi^{2}$-test | Problem | No Problem | $\chi^{2}$-test | Problem | No Problem | $\chi^{2}$-test |
|  | % | % | p-value | % | % | p-value | % | % | p-value | % | % | p-value |
| Managing authority |  |  |  |  |  |  |  |  |  |  |  |  |
| Private | 9.7 | 16.8 | 0.080 | 21.4 | 15.7 | 0.416 | 4.7 | 17.7 | **0.001***** | 11.1 | 16.2 | 0.364 |
| Public | 90.3 | 83.2 |  | 78.6 | 84.3 |  | 95.3 | 82.3 |  | 88.9 | 83.8 |  |
| Contraceptive stocks |  |  |  |  |  |  |  |  |  |  |  |  |
| In-stock | 76.3 | 88.4 | **0.001***** | 89.3 | 86.8 | 0.707 | 92.5 | 86.0 | **0.065*** | 93.3 | 86.5 | 0.189 |
| Not offered/Out-of-stock | 23.7 | 11.6 |  | 10.7 | 13.2 |  | 7.5 | 14.0 |  | 6.7 | 13.5 |  |
| Fee for FP methods |  |  |  |  |  |  |  |  |  |  |  |  |
| No | 76.3 | 55.6 | **0.000***** | 71.4 | 57.6 | 0.145 | 68.2 | 56.5 | **0.022**** | 48.9 | 58.7 | 0.198 |
| Yes | 23.7 | 44.4 |  | 28.6 | 42.4 |  | 31.8 | 43.5 |  | 51.1 | 41.3 |  |
| Provider discussed FP |  |  |  |  |  |  |  |  |  |  |  |  |
| No | 21.1 | 22.1 | 0.820 | 21.4 | 22.0 | 0.945 | 21.3 | 22.1 | 0.858 | 28.3 | 21.6 | 0.287 |
| Yes | 78.9 | 77.9 |  | 78.6 | 78.0 |  | 78.7 | 77.9 |  | 71.7 | 78.4 |  |
| Used the baseline method before the visit |  |  |  |  |  |  |  |  |  |  |  |  |
| No | 27.4 | 27.8 | 0.933 | 35.7 | 27.4 | 0.337 | 36.1 | 26.4 | **0.036**** | 23.9 | 28 | 0.551 |
| Yes | 72.6 | 72.2 |  | 64.3 | 72.6 |  | 63.9 | 73.6 |  | 76.1 | 72 |  |
| Baseline method |  |  |  |  |  |  |  |  |  |  |  |  |
| Long-acting reversible contraception | 29.5 | 31.3 | 0.712 | 53.6 | 30.3 | **0.009**** | 37 | 30.2 | 0.153 | 39.1 | 30.6 | 0.227 |
| Short-acting reversible contraception | 70.5 | 68.7 |  | 46.4 | 69.7 |  | 63 | 69.8 |  | 60.9 | 69.4 |  |
| Heard FP in radio or TV (last 3 months) |  |  |  |  |  |  |  |  |  |  |  |  |
| No | 23.4 | 30.1 | 0.181 | 17.9 | 29.7 | 0.175 | 27.8 | 29.5 | 0.707 | 43.5 | 28.4 | **0.030**** |
| Yes | 76.6 | 69.9 |  | 82.1 | 70.3 |  | 72.2 | 70.5 |  | 56.5 | 71.6 |  |
| CHW talked about FP recently |  |  |  |  |  |  |  |  |  |  |  |  |
| No | 86.2 | 87.2 | 0.788 | 82.1 | 87.2 | 0.432 | 91.6 | 86.3 | 0.132 | 84.8 | 87.2 | 0.638 |
| Yes | 13.8 | 12.8 |  | 17.9 | 12.8 |  | 8.4 | 13.7 |  | 15.2 | 12.8 |  |
| Marital status |  |  |  |  |  |  |  |  |  |  |  |  |
| In union | 86.3 | 90.9 | 0.157 | 85.7 | 90.5 | 0.399 | 88.9 | 90.6 | 0.583 | 89.1 | 90.4 | 0.775 |
| Not in union | 13.7 | 9.1 |  | 14.3 | 9.5 |  | 11.1 | 9.4 |  | 10.9 | 9.6 |  |
| Age |  |  |  |  |  |  |  |  |  |  |  |  |
| 18-24 | 21.1 | 26.1 | 0.357 | 17.9 | 25.7 | 0.155 | 21.3 | 26.1 | 0.326 | 21.7 | 25.7 | 0.758 |
| 25-34 | 56.8 | 49.1 |  | 67.9 | 49.4 |  | 49.1 | 50.2 |  | 50.0 | 50.1 |  |
| 35-49 | 22.1 | 24.8 |  | 14.3 | 24.8 |  | 29.6 | 23.7 |  | 28.3 | 24.2 |  |
| Parity |  |  |  |  |  |  |  |  |  |  |  |  |
| None-1 | 21.1 | 23.4 | 0.428 | 25.0 | 23.0 | **0.032**** | 22.2 | 23.2 | 0.867 | 23.9 | 23.0 | 0.105 |
| 2-3 | 54.7 | 47.7 |  | 67.9 | 47.9 |  | 50.9 | 48.2 |  | 60.9 | 47.8 |  |
| 4+ | 24.2 | 28.9 |  | 7.1 | 29.1 |  | 26.9 | 28.6 |  | 15.2 | 29.2 |  |
| Education |  |  |  |  |  |  |  |  |  |  |  |  |
| None/primary | 33.7 | 33.3 | 0.117 | 32.1 | 33.4 | 0.983 | 39.8 | 32.4 | 0.134 | 37.0 | 33.2 | 0.204 |
| Post-primary/Secondary | 53.7 | 45.4 |  | 46.4 | 46.4 |  | 46.3 | 46.4 |  | 34.8 | 47.1 |  |
| College/University | 12.6 | 21.2 |  | 21.4 | 20.2 |  | 13.9 | 21.2 |  | 28.3 | 19.7 |  |
| Wealth |  |  |  |  |  |  |  |  |  |  |  |  |
| Poorest | 32.6 | 38.3 | 0.342 | 25.0 | 38.0 | 0.283 | 43.0 | 36.7 | 0.421 | 45.7 | 37.1 | 0.458 |
| Middle | 56.8 | 48.9 |  | 64.3 | 49.3 |  | 46.7 | 50.3 |  | 41.3 | 50.3 |  |
| Richest | 10.5 | 12.9 |  | 10.7 | 12.7 |  | 10.3 | 13.0 |  | 13.0 | 12.6 |  |
| Discussed FP w/ partner (last 6 months) |  |  |  |  |  |  |  |  |  |  |  |  |
| No | 45.3 | 32.1 | **0.011**** | 42.9 | 33.3 | 0.295 | 43.5 | 32.1 | **0.020**** | 47.8 | 32.8 | **0.036**** |
| Yes | 54.7 | 67.9 |  | 57.1 | 66.7 |  | 56.5 | 67.9 |  | 52.2 | 67.2 |  |
| Notes: Bold values indicate significant differences. *** p<0.01, ** p<0.05, * p<0.1. | | | | | | | | | | | | |

# Table A- 4: Results from probit regression models of client dissatisfaction with family planning services on contraceptive discontinuation

| Variables | Outcome: Contraceptive Discontinuation | | | | | | | | | |
| --- | --- | --- | --- | --- | --- | --- | --- | --- | --- | --- |
|  | Model 1 | | Model 2 | | Model 3 | | Model 4 | | Model 5 | |
|  | Coeff. | Avg. Marg. Effects | Coeff. | Avg. Marg. Effects | Coeff. | Avg. Marg. Effects | Coeff. | Avg. Marg. Effects | Coeff. | Avg. Marg. Effects |
| **Reports problem w/ availability *(ref. No)*** |  |  |  |  |  |  |  |  |  |  |
| Yes | 0.137 | 0.033 | 0.134 | 0.032 |  |  |  |  |  |  |
|  | [-0.228 - 0.502] | [-0.055 - 0.121] | [-0.212 - 0.479] | [-0.051 - 0.116] |  |  |  |  |  |  |
| **Reports problem w/ privacy *(ref. No)*** |  |  |  |  |  |  |  |  |  |  |
| Yes | -0.372 | -0.090 |  |  | -0.266 | -0.065 |  |  |  |  |
|  | [-0.982 - 0.239] | [-0.237 - 0.057] |  |  | [-0.855 - 0.323] | [-0.207 - 0.078] |  |  |  |  |
| **Reports problem w/ convenience *(ref. No)*** |  |  |  |  |  |  |  |  |  |  |
| Yes | 0.003 | 0.001 |  |  |  |  | 0.031 | 0.007 |  |  |
|  | [-0.342 - 0.348] | [-0.083 - 0.084] |  |  |  |  | [-0.297 - 0.359] | [-0.072 - 0.087] |  |  |
| **Reports problem w/ clean/staff treatment *(ref. No)*** |  |  |  |  |  |  |  |  |  |  |
| Yes | **0.341*** | **0.082*** |  |  |  |  |  |  | 0.320 | 0.078 |
|  | [-0.056 - 0.737] | [-0.013 - 0.178] |  |  |  |  |  |  | [-0.071 - 0.711] | [-0.017 - 0.172] |
| **Managing authority (*ref. Private*)** |  |  |  |  |  |  |  |  |  |  |
| Public | -0.168 | -0.043 | -0.169 | -0.043 | -0.156 | -0.039 | -0.161 | -0.041 | -0.158 | -0.040 |
|  | [-0.517 - 0.180] | [-0.135 - 0.049] | [-0.514 - 0.175] | [-0.135 - 0.048] | [-0.498 - 0.187] | [-0.130 - 0.051] | [-0.509 - 0.187] | [-0.133 - 0.051] | [-0.501 - 0.184] | [-0.130 - 0.050] |
| **Contraceptive stocks (*ref. In-stock*)** |  |  |  |  |  |  |  |  |  |  |
| Not offered/Out-of-stock | 0.118 | 0.030 | 0.111 | 0.028 | 0.129 | 0.033 | 0.129 | 0.033 | 0.135 | 0.034 |
|  | [-0.211 - 0.447] | [-0.056 - 0.115] | [-0.217 - 0.440] | [-0.057 - 0.113] | [-0.196 - 0.455] | [-0.053 - 0.118] | [-0.197 - 0.456] | [-0.053 - 0.118] | [-0.192 - 0.462] | [-0.052 - 0.120] |
| **Fee for FP methods (*ref. No*)** |  |  |  |  |  |  |  |  |  |  |
| Yes | 0.153 | 0.037 | 0.150 | 0.037 | 0.148 | 0.036 | 0.148 | 0.036 | 0.147 | 0.036 |
|  | [-0.147 - 0.453] | [-0.037 - 0.111] | [-0.149 - 0.449] | [-0.037 - 0.111] | [-0.150 - 0.447] | [-0.037 - 0.110] | [-0.151 - 0.446] | [-0.038 - 0.110] | [-0.152 - 0.447] | [-0.038 - 0.110] |
| **Provider discussed FP (*ref. No*)** |  |  |  |  |  |  |  |  |  |  |
| Yes | **-0.290**** | **-0.075**** | **-0.304**** | **-0.079**** | **-0.297**** | **-0.077**** | **-0.300**** | **-0.078**** | **-0.292**** | **-0.076**** |
|  | [-0.550 - -0.030] | [-0.146 - -0.004] | [-0.564 - -0.043] | [-0.151 - -0.007] | [-0.557 - -0.037] | [-0.149 - -0.006] | [-0.561 - -0.040] | [-0.150 - -0.007] | [-0.552 - -0.033] | [-0.147 - -0.005] |
| **Used the baseline method before the visit (*ref. No*)** |  |  |  |  |  |  |  |  |  |  |
| Yes | 0.101 | 0.024 | 0.113 | 0.027 | 0.111 | 0.026 | 0.114 | 0.027 | 0.104 | 0.025 |
|  | [-0.168 - 0.370] | [-0.039 - 0.087] | [-0.154 - 0.379] | [-0.035 - 0.089] | [-0.156 - 0.378] | [-0.036 - 0.088] | [-0.155 - 0.383] | [-0.035 - 0.090] | [-0.164 - 0.371] | [-0.038 - 0.087] |
| **Baseline method (*ref. LARC*)** |  |  |  |  |  |  |  |  |  |  |
| Short-acting reversible contraception | **-0.412***** | **-0.107***** | **-0.409***** | **-0.106***** | **-0.420***** | **-0.109***** | **-0.409***** | **-0.106***** | **-0.399***** | **-0.103***** |
|  | [-0.656 - -0.169] | [-0.172 - -0.041] | [-0.653 - -0.164] | [-0.172 - -0.040] | [-0.664 - -0.177] | [-0.175 - -0.043] | [-0.653 - -0.165] | [-0.172 - -0.040] | [-0.643 - -0.155] | [-0.169 - -0.037] |
| **Heard FP in radio or TV (last 3 months) (*ref. No*)** |  |  |  |  |  |  |  |  |  |  |
| Yes | -0.018 | -0.004 | -0.031 | -0.007 | -0.029 | -0.007 | -0.030 | -0.007 | -0.022 | -0.005 |
|  | [-0.263 - 0.227] | [-0.064 - 0.055] | [-0.274 - 0.213] | [-0.067 - 0.052] | [-0.272 - 0.215] | [-0.067 - 0.053] | [-0.273 - 0.213] | [-0.067 - 0.052] | [-0.267 - 0.223] | [-0.065 - 0.055] |
| **CHW talked about FP recently (*ref. No*)** |  |  |  |  |  |  |  |  |  |  |
| Yes | 0.034 | 0.008 | 0.038 | 0.009 | 0.036 | 0.009 | 0.041 | 0.010 | 0.038 | 0.009 |
|  | [-0.295 - 0.363] | [-0.073 - 0.090] | [-0.289 - 0.364] | [-0.072 - 0.090] | [-0.290 - 0.362] | [-0.072 - 0.090] | [-0.288 - 0.370] | [-0.072 - 0.092] | [-0.289 - 0.365] | [-0.072 - 0.090] |
| **Marital status (*ref. In union*)** |  |  |  |  |  |  |  |  |  |  |
| Not in union | 0.001 | 0.000 | 0.002 | 0.001 | 0.008 | 0.002 | 0.005 | 0.001 | -0.002 | -0.000 |
|  | [-0.387 - 0.390] | [-0.094 - 0.094] | [-0.388 - 0.392] | [-0.094 - 0.095] | [-0.380 - 0.397] | [-0.093 - 0.097] | [-0.384 - 0.394] | [-0.094 - 0.096] | [-0.390 - 0.386] | [-0.095 - 0.094] |
| **Age (*ref. 18-24*)** |  |  |  |  |  |  |  |  |  |  |
| 25-34 | **0.285*** | **0.070**** | **0.277*** | **0.069**** | **0.293**** | **0.072**** | **0.282*** | **0.070**** | **0.279*** | **0.069**** |
|  | [-0.007 - 0.576] | [0.002 - 0.138] | [-0.016 - 0.570] | [0.000 - 0.137] | [0.003 - 0.584] | [0.005 - 0.140] | [-0.009 - 0.573] | [0.002 - 0.138] | [-0.013 - 0.571] | [0.001 - 0.137] |
| 35-49 | -0.110 | -0.023 | -0.108 | -0.023 | -0.092 | -0.019 | -0.104 | -0.022 | -0.114 | -0.024 |
|  | [-0.510 - 0.291] | [-0.105 - 0.060] | [-0.507 - 0.290] | [-0.106 - 0.060] | [-0.486 - 0.303] | [-0.101 - 0.063] | [-0.502 - 0.293] | [-0.104 - 0.061] | [-0.512 - 0.285] | [-0.106 - 0.059] |
| **Parity (*ref. None-1*)** |  |  |  |  |  |  |  |  |  |  |
| 2-3 | **-0.538***** | **-0.147***** | **-0.537***** | **-0.148***** | **-0.530***** | **-0.146***** | **-0.532***** | **-0.147***** | **-0.537***** | **-0.147***** |
|  | [-0.836 - -0.239] | [-0.236 - -0.059] | [-0.836 - -0.238] | [-0.237 - -0.059] | [-0.827 - -0.232] | [-0.235 - -0.058] | [-0.831 - -0.234] | [-0.236 - -0.058] | [-0.835 - -0.238] | [-0.236 - -0.059] |
| 4+ | **-0.579***** | **-0.156***** | **-0.578***** | **-0.157***** | **-0.594***** | **-0.160***** | **-0.580***** | **-0.157***** | **-0.566***** | **-0.154***** |
|  | [-0.975 - -0.184] | [-0.264 - -0.049] | [-0.972 - -0.183] | [-0.264 - -0.049] | [-0.986 - -0.201] | [-0.267 - -0.054] | [-0.974 - -0.187] | [-0.264 - -0.050] | [-0.961 - -0.171] | [-0.261 - -0.046] |
| **Education (*ref. None/primary*)** |  |  |  |  |  |  |  |  |  |  |
| Post-Primary/Secondary | **-0.352***** | **-0.089***** | **-0.351***** | **-0.090***** | **-0.351***** | **-0.090***** | **-0.346***** | **-0.088***** | **-0.339***** | **-0.086**** |
|  | [-0.607 - -0.097] | [-0.156 - -0.023] | [-0.606 - -0.097] | [-0.156 - -0.024] | [-0.604 - -0.097] | [-0.155 - -0.024] | [-0.600 - -0.091] | [-0.154 - -0.022] | [-0.594 - -0.084] | [-0.153 - -0.020] |
| College/University | **-0.391**** | **-0.098**** | **-0.383**** | **-0.096**** | **-0.387**** | **-0.097**** | **-0.383**** | **-0.096**** | **-0.386**** | **-0.097**** |
|  | [-0.731 - -0.050] | [-0.179 - -0.017] | [-0.722 - -0.043] | [-0.178 - -0.015] | [-0.726 - -0.049] | [-0.179 - -0.016] | [-0.722 - -0.043] | [-0.178 - -0.015] | [-0.727 - -0.045] | [-0.178 - -0.015] |
| **Wealth (*ref. Poorest (1-3)*)** |  |  |  |  |  |  |  |  |  |  |
| Middle (4-5) | -0.119 | -0.029 | -0.127 | -0.032 | -0.121 | -0.030 | -0.123 | -0.030 | -0.120 | -0.030 |
|  | [-0.364 - 0.126] | [-0.090 - 0.032] | [-0.372 - 0.118] | [-0.093 - 0.030] | [-0.367 - 0.125] | [-0.092 - 0.031] | [-0.368 - 0.123] | [-0.092 - 0.031] | [-0.366 - 0.126] | [-0.091 - 0.032] |
| Richest (6-10) | -0.215 | -0.051 | -0.213 | -0.051 | -0.217 | -0.052 | -0.214 | -0.051 | -0.213 | -0.051 |
|  | [-0.599 - 0.170] | [-0.138 - 0.036] | [-0.598 - 0.171] | [-0.139 - 0.037] | [-0.600 - 0.167] | [-0.139 - 0.036] | [-0.598 - 0.171] | [-0.139 - 0.037] | [-0.598 - 0.173] | [-0.138 - 0.037] |
| **Discussed FP w/ partner (last 6 months) (*ref. No*)** |  |  |  |  |  |  |  |  |  |  |
| Yes | **-0.236*** | **-0.059*** | **-0.242*** | **-0.061*** | **-0.250*** | **-0.063*** | **-0.246*** | **-0.062*** | **-0.237*** | **-0.060*** |
|  | [-0.491 - 0.020] | [-0.125 - 0.007] | [-0.496 - 0.013] | [-0.127 - 0.005] | [-0.503 - 0.004] | [-0.129 - 0.003] | [-0.500 - 0.007] | [-0.128 - 0.004] | [-0.491 - 0.017] | [-0.125 - 0.006] |
| Constant | 0.157 |  | 0.173 |  | 0.192 |  | 0.170 |  | 0.140 |  |
|  | [-0.415 - 0.728] |  | [-0.396 - 0.741] |  | [-0.376 - 0.760] |  | [-0.401 - 0.742] |  | [-0.428 - 0.709] |  |
| Observations | 765 |  | 765 |  | 765 |  | 765 |  | 765 |  |
| Notes: Reporting probit coefficients (Coeff.) with 95% CIs computed with robust standard errors in brackets. Reporting average marginal effects (Avg. Marg. Effects) with CIs computed with the delta-method in brackets. All models control for country fixed effects. *** p<0.01, ** p<0.05, * p<0.1. | | | | | | | | | | |

# Table A- 5: Regression results from the univariate probit

| Variables | Discontinuer | | | | | | | | | | | | |
| --- | --- | --- | --- | --- | --- | --- | --- | --- | --- | --- | --- | --- | --- |
| Time waited to see a provider = problem | 0.400 | 0.087 |  |  |  |  |  |  |  |  |  |  |  |
|  | [-0.412 - 0.812] | [-0.301 - 0.475] |  |  |  |  |  |  |  |  |  |  |  |
| Amount of explanation received = problem | 0.282 |  | 0.289 |  |  |  |  |  |  |  |  |  |  |
|  | [-0.375 - 0.939] |  | [-0.285 - 0.864] |  |  |  |  |  |  |  |  |  |  |
| Visual privacy during examination = problem | -0.157 |  |  | -0.157 |  |  |  |  |  |  |  |  |  |
|  | [-0.980 - 0.665] |  |  | [-0.768 - 0.454] |  |  |  |  |  |  |  |  |  |
| Auditory privacy during examination = problem | -0.258 |  |  |  | -0.418 |  |  |  |  |  |  |  |  |
|  | [-1.273 - 0.756] |  |  |  | [-1.224 - 0.387] |  |  |  |  |  |  |  |  |
| Range of services available = problem | -0.081 |  |  |  |  | 0.053 |  |  |  |  |  |  |  |
|  | [-0.642 - 0.480] |  |  |  |  | [-0.411 - 0.518] |  |  |  |  |  |  |  |
| Availability of medicines = problem | 0.092 |  |  |  |  |  | 0.164 |  |  |  |  |  |  |
|  | [-0.384 - 0.569] |  |  |  |  |  | [-0.260 - 0.588] |  |  |  |  |  |  |
| Availability of contraceptives = problem | 0.233 |  |  |  |  |  |  | 0.258 |  |  |  |  |  |
|  | [-0.316 - 0.782] |  |  |  |  |  |  | [-0.237 - 0.752] |  |  |  |  |  |
| Hours of Service = problem | 0.287 |  |  |  |  |  |  |  | 0.221 |  |  |  |  |
|  | [-0.252 - 0.827] |  |  |  |  |  |  |  | [-0.282 - 0.724] |  |  |  |  |
| Days of service = problem | -0.302 |  |  |  |  |  |  |  |  | -0.088 |  |  |  |
|  | [-1.007 - 0.404] |  |  |  |  |  |  |  |  | [-0.672 - 0.495] |  |  |  |
| Cleanliness of the facility = problem | 0.373 |  |  |  |  |  |  |  |  |  | 0.335 |  |  |
|  | [-0.111 - 0.858] |  |  |  |  |  |  |  |  |  | [-0.106 - 0.776] |  |  |
| Staff treatment = problem | 0.106 |  |  |  |  |  |  |  |  |  |  | 0.255 |  |
|  | [-0.548 - 0.759] |  |  |  |  |  |  |  |  |  |  | [-0.414 - 0.924] |  |
| Cost of services or treatment = problem | 0.011 |  |  |  |  |  |  |  |  |  |  |  | 0.05 |
|  | [-0.533 - 0.555] |  |  |  |  |  |  |  |  |  |  |  | [-0.471 - 0.572] |
| Managing authority = 1, Public | -0.209 | -0.163 | -0.149 | -0.156 | -0.16 | -0.197 | -0.188 | -0.17 | -0.168 | -0.157 | -0.169 | -0.151 | -0.152 |
|  | [-0.569 - 0.150] | [-0.509 - 0.184] | [-0.492 - 0.193] | [-0.498 - 0.186] | [-0.502 - 0.182] | [-0.546 - 0.151] | [-0.535 - 0.159] | [-0.514 - 0.174] | [-0.516 - 0.180] | [-0.501 - 0.187] | [-0.512 - 0.174] | [-0.493 - 0.192] | [-0.494 - 0.190] |
| Stock of contraception = 1, Not offered/Out-of-stock | 0.09 | 0.13 | 0.132 | 0.129 | 0.134 | 0.116 | 0.066 | 0.118 | 0.141 | 0.135 | 0.132 | 0.132 | 0.128 |
|  | [-0.239 - 0.419] | [-0.196 - 0.456] | [-0.194 - 0.458] | [-0.197 - 0.455] | [-0.192 - 0.459] | [-0.212 - 0.444] | [-0.265 - 0.396] | [-0.209 - 0.445] | [-0.186 - 0.468] | [-0.192 - 0.462] | [-0.195 - 0.460] | [-0.194 - 0.458] | [-0.197 - 0.454] |
| Clients charged for FP methods = 1 | 0.188 | 0.149 | 0.158 | 0.148 | 0.147 | 0.147 | 0.171 | 0.151 | 0.156 | 0.148 | 0.139 | 0.154 | 0.149 |
|  | [-0.118 - 0.494] | [-0.149 - 0.447] | [-0.139 - 0.455] | [-0.151 - 0.446] | [-0.151 - 0.445] | [-0.150 - 0.444] | [-0.131 - 0.473] | [-0.147 - 0.449] | [-0.142 - 0.455] | [-0.151 - 0.447] | [-0.160 - 0.438] | [-0.145 - 0.453] | [-0.152 - 0.449] |
| Did your provider discuss family planning with you during your visit today? = 1, Yes | **-0.304**** | **-0.301**** | **-0.299**** | **-0.298**** | **-0.293**** | **-0.297**** | **-0.300**** | **-0.301**** | **-0.306**** | **-0.319**** | **-0.298**** | **-0.294**** | **-0.299**** |
|  | [-0.566 - -0.042] | [-0.561 - -0.041] | [-0.558 - -0.040] | [-0.558 - -0.038] | [-0.553 - -0.033] | [-0.556 - -0.037] | [-0.560 - -0.040] | [-0.561 - -0.041] | [-0.568 - -0.044] | [-0.579 - -0.058] | [-0.559 - -0.038] | [-0.553 - -0.035] | [-0.559 - -0.039] |
| Used a method before the visit = 1, New user | 0.105 | 0.111 | 0.12 | 0.112 | 0.119 | 0.1 | 0.105 | 0.113 | 0.114 | 0.115 | 0.108 | 0.11 | 0.113 |
|  | [-0.170 - 0.379] | [-0.157 - 0.380] | [-0.148 - 0.387] | [-0.156 - 0.379] | [-0.148 - 0.386] | [-0.167 - 0.368] | [-0.163 - 0.373] | [-0.154 - 0.380] | [-0.158 - 0.386] | [-0.152 - 0.383] | [-0.159 - 0.375] | [-0.157 - 0.377] | [-0.154 - 0.381] |
| Baseline method = 2, STMs | **-0.421***** | **-0.405***** | **-0.397***** | **-0.416***** | **-0.419***** | **-0.421***** | **-0.420***** | **-0.408***** | **-0.407***** | **-0.416***** | **-0.405***** | **-0.403***** | **-0.407***** |
|  | [-0.672 - -0.170] | [-0.650 - -0.160] | [-0.643 - -0.151] | [-0.659 - -0.172] | [-0.663 - -0.175] | [-0.665 - -0.177] | [-0.665 - -0.174] | [-0.652 - -0.163] | [-0.654 - -0.160] | [-0.661 - -0.171] | [-0.649 - -0.161] | [-0.648 - -0.159] | [-0.651 - -0.163] |
| In the past 3-months heard about FP on the radio or TV = 1, Yes | -0.028 | -0.031 | -0.041 | -0.029 | -0.032 | -0.031 | -0.015 | -0.035 | -0.026 | -0.029 | -0.024 | -0.028 | -0.034 |
|  | [-0.276 - 0.220] | [-0.274 - 0.212] | [-0.285 - 0.202] | [-0.272 - 0.214] | [-0.275 - 0.211] | [-0.275 - 0.212] | [-0.261 - 0.231] | [-0.278 - 0.209] | [-0.269 - 0.218] | [-0.272 - 0.215] | [-0.268 - 0.221] | [-0.272 - 0.215] | [-0.277 - 0.210] |
| In the last 12 months, were you visited by a CHW who talked to you about FP? = 1, Yes | 0.076 | 0.041 | 0.047 | 0.036 | 0.042 | 0.03 | 0.035 | 0.044 | 0.071 | 0.043 | 0.044 | 0.036 | 0.037 |
|  | [-0.258 - 0.410] | [-0.286 - 0.369] | [-0.279 - 0.374] | [-0.290 - 0.362] | [-0.285 - 0.369] | [-0.296 - 0.356] | [-0.292 - 0.363] | [-0.283 - 0.371] | [-0.259 - 0.400] | [-0.285 - 0.371] | [-0.283 - 0.371] | [-0.291 - 0.362] | [-0.289 - 0.363] |
| Marital Status = 2, Not in Union | 0.032 | 0.005 | 0.015 | 0.008 | 0.007 | 0.002 | 0.011 | -0.001 | 0.024 | -0.002 | -0.001 | 0.004 | 0.006 |
|  | [-0.363 - 0.426] | [-0.385 - 0.394] | [-0.375 - 0.405] | [-0.381 - 0.397] | [-0.381 - 0.395] | [-0.388 - 0.392] | [-0.381 - 0.402] | [-0.392 - 0.390] | [-0.365 - 0.412] | [-0.391 - 0.388] | [-0.389 - 0.388] | [-0.385 - 0.393] | [-0.383 - 0.395] |
| Age group = 2, 25-34 | **0.271*** | **0.277*** | **0.284*** | **0.289*** | **0.292**** | **0.276*** | **0.282*** | **0.278*** | **0.270*** | **0.284*** | **0.280*** | **0.281*** | **0.286*** |
|  | [-0.028 - 0.569] | [-0.015 - 0.569] | [-0.009 - 0.578] | [-0.002 - 0.580] | [0.001 - 0.583] | [-0.015 - 0.568] | [-0.012 - 0.575] | [-0.015 - 0.570] | [-0.023 - 0.564] | [-0.009 - 0.576] | [-0.012 - 0.572] | [-0.011 - 0.572] | [-0.006 - 0.578] |
| Age group = 3, 35-49 | -0.075 | -0.11 | -0.093 | -0.096 | -0.095 | -0.104 | -0.074 | -0.114 | -0.114 | -0.096 | -0.103 | -0.11 | -0.098 |
|  | [-0.478 - 0.329] | [-0.508 - 0.287] | [-0.491 - 0.305] | [-0.491 - 0.300] | [-0.490 - 0.300] | [-0.499 - 0.292] | [-0.470 - 0.322] | [-0.512 - 0.283] | [-0.511 - 0.283] | [-0.494 - 0.302] | [-0.500 - 0.294] | [-0.508 - 0.287] | [-0.495 - 0.298] |
| Parity group = 2, 2-3 | **-0.531***** | **-0.530***** | **-0.530***** | **-0.531***** | **-0.537***** | **-0.533***** | **-0.546***** | **-0.543***** | **-0.507***** | **-0.531***** | **-0.535***** | **-0.535***** | **-0.533***** |
|  | [-0.839 - -0.223] | [-0.829 - -0.231] | [-0.829 - -0.231] | [-0.828 - -0.233] | [-0.835 - -0.238] | [-0.832 - -0.235] | [-0.847 - -0.244] | [-0.843 - -0.243] | [-0.807 - -0.206] | [-0.829 - -0.232] | [-0.833 - -0.236] | [-0.834 - -0.237] | [-0.833 - -0.233] |
| Parity group = 3, 4+ | **-0.600***** | **-0.578***** | **-0.574***** | **-0.588***** | **-0.595***** | **-0.577***** | **-0.612***** | **-0.581***** | **-0.561***** | **-0.584***** | **-0.569***** | **-0.577***** | **-0.582***** |
|  | [-0.998 - -0.203] | [-0.971 - -0.184] | [-0.968 - -0.179] | [-0.981 - -0.195] | [-0.988 - -0.202] | [-0.970 - -0.184] | [-1.005 - -0.219] | [-0.975 - -0.186] | [-0.956 - -0.166] | [-0.977 - -0.190] | [-0.964 - -0.175] | [-0.971 - -0.183] | [-0.976 - -0.189] |
| Grouped schooling level = 2, Post-Primary/Secondary | **-0.359***** | **-0.349***** | **-0.337***** | **-0.349***** | **-0.350***** | **-0.345***** | **-0.362***** | **-0.343***** | **-0.350***** | **-0.351***** | **-0.340***** | **-0.342***** | **-0.346***** |
|  | [-0.617 - -0.101] | [-0.603 - -0.095] | [-0.590 - -0.083] | [-0.602 - -0.096] | [-0.604 - -0.096] | [-0.599 - -0.090] | [-0.619 - -0.105] | [-0.597 - -0.089] | [-0.605 - -0.095] | [-0.606 - -0.097] | [-0.594 - -0.085] | [-0.597 - -0.088] | [-0.600 - -0.092] |
| Grouped schooling level = 3, College/University | **-0.401**** | **-0.384**** | **-0.371**** | **-0.385**** | **-0.394**** | **-0.395**** | **-0.417**** | **-0.379**** | **-0.362**** | **-0.389**** | **-0.386**** | **-0.383**** | **-0.379**** |
|  | [-0.754 - -0.047] | [-0.723 - -0.044] | [-0.711 - -0.030] | [-0.724 - -0.047] | [-0.732 - -0.055] | [-0.738 - -0.052] | [-0.763 - -0.070] | [-0.719 - -0.039] | [-0.703 - -0.021] | [-0.729 - -0.049] | [-0.727 - -0.046] | [-0.723 - -0.043] | [-0.719 - -0.040] |
| Recoded HH ladder_v2 = 2, Middle 4-5 | -0.109 | -0.12 | -0.126 | -0.123 | -0.118 | -0.115 | -0.118 | -0.131 | -0.129 | -0.126 | -0.119 | -0.124 | -0.125 |
|  | [-0.360 - 0.142] | [-0.366 - 0.125] | [-0.374 - 0.121] | [-0.368 - 0.123] | [-0.363 - 0.128] | [-0.361 - 0.131] | [-0.364 - 0.128] | [-0.376 - 0.114] | [-0.376 - 0.118] | [-0.373 - 0.120] | [-0.365 - 0.127] | [-0.370 - 0.122] | [-0.373 - 0.122] |
| Recoded HH ladder_v2 = 3, Richest 6-10 | -0.304 | -0.21 | -0.225 | -0.216 | -0.215 | -0.192 | -0.238 | -0.217 | -0.269 | -0.214 | -0.209 | -0.215 | -0.22 |
|  | [-0.711 - 0.103] | [-0.595 - 0.176] | [-0.610 - 0.160] | [-0.600 - 0.168] | [-0.599 - 0.169] | [-0.580 - 0.196] | [-0.632 - 0.156] | [-0.601 - 0.167] | [-0.663 - 0.125] | [-0.600 - 0.171] | [-0.594 - 0.176] | [-0.599 - 0.170] | [-0.604 - 0.165] |
| In the last 6-months have you discussed FP with your partner? = 1, Yes | **-0.219*** | **-0.248*** | **-0.242*** | **-0.248*** | **-0.250*** | **-0.244*** | **-0.234*** | **-0.237*** | **-0.246*** | **-0.239*** | **-0.235*** | **-0.248*** | **-0.247*** |
|  | [-0.478 - 0.040] | [-0.502 - 0.006] | [-0.496 - 0.011] | [-0.501 - 0.005] | [-0.504 - 0.003] | [-0.496 - 0.008] | [-0.488 - 0.021] | [-0.491 - 0.017] | [-0.502 - 0.010] | [-0.493 - 0.016] | [-0.489 - 0.018] | [-0.501 - 0.005] | [-0.500 - 0.006] |
| iso3 = 566, Nigeria | **0.377**** | **0.351**** | **0.340**** | **0.341**** | **0.338**** | **0.366**** | **0.363**** | **0.347**** | **0.365**** | **0.328**** | **0.348**** | **0.336**** | **0.345**** |
|  | [0.039 - 0.716] | [0.030 - 0.671] | [0.024 - 0.656] | [0.026 - 0.657] | [0.023 - 0.654] | [0.044 - 0.687] | [0.039 - 0.687] | [0.029 - 0.666] | [0.040 - 0.689] | [0.011 - 0.645] | [0.031 - 0.665] | [0.020 - 0.653] | [0.028 - 0.661] |
| iso3 = 854, Burkina Faso | 0.129 | 0.147 | 0.146 | 0.135 | 0.13 | 0.158 | 0.147 | 0.158 | 0.154 | 0.131 | 0.139 | 0.14 | 0.139 |
|  | [-0.262 - 0.521] | [-0.228 - 0.523] | [-0.229 - 0.520] | [-0.241 - 0.512] | [-0.246 - 0.505] | [-0.221 - 0.537] | [-0.232 - 0.526] | [-0.221 - 0.537] | [-0.228 - 0.536] | [-0.245 - 0.507] | [-0.238 - 0.515] | [-0.235 - 0.515] | [-0.236 - 0.514] |
| Constant | 0.174 | 0.17 | 0.134 | 0.183 | 0.192 | 0.215 | 0.195 | 0.177 | 0.151 | 0.203 | 0.154 | 0.159 | 0.169 |
|  | [-0.414 - 0.762] | [-0.398 - 0.739] | [-0.438 - 0.707] | [-0.384 - 0.751] | [-0.376 - 0.759] | [-0.357 - 0.786] | [-0.377 - 0.768] | [-0.390 - 0.745] | [-0.422 - 0.724] | [-0.370 - 0.776] | [-0.414 - 0.721] | [-0.410 - 0.728] | [-0.399 - 0.737] |
|  |  |  |  |  |  |  |  |  |  |  |  |  |  |
| Observations | 745 | 764 | 763 | 765 | 765 | 761 | 757 | 764 | 757 | 761 | 765 | 765 | 764 |

# Table A- 6: Percentage of clients who discontinued contraception and experienced problems during a family planning visit by country

| Country | Obs. | Discontinuer | Problem w/ Availability | Problem w/ Privacy | Problem w/ Convenience | Problem w/ Clean/Staff treatment |
| --- | --- | --- | --- | --- | --- | --- |
| Kenya | 330 | 16.7 | 18.5 | 6.4 | 18.5 | 3.9 |
| Nigeria | 260 | 16.5 | 6.5 | 2.3 | 5.0 | 4.2 |
| Burkina Faso | 207 | 22.7 | 8.2 | 0.5 | 16.4 | 10.6 |
|  |  |  |  |  |  |  |

# Table A- 7: Percentage of clients who discontinued contraception by method type used in the baseline interview

| Method type | | Continuer | Discontinuer | Total |
| --- | --- | --- | --- | --- |
| IUD | Obs. | 41 | 20 | 61 |
|  | % | 67.21 | 32.79 | 100 |
| Implants | Obs. | 146 | 41 | 187 |
|  | % | 78.07 | 21.93 | 100 |
| Injectables | Obs. | 408 | 63 | 471 |
|  | % | 86.62 | 13.38 | 100 |
| Pill | Obs. | 45 | 19 | 64 |
|  | % | 70.31 | 29.69 | 100 |
| Male Condom | Obs. | 9 | 2 | 11 |
|  | % | 81.82 | 18.18 | 100 |
| Other | Obs. | 3 | 0 | 3 |
|  | % | 100 | 0 | 100 |
| Total | Obs. | 652 | 145 | 797 |
|  | % | 81.81 | 18.19 | 100 |

# Table A- 8: List of variables used in the analysis

| Variable | Type | CEI questionnaire | SDP questionnaire | Notes |
| --- | --- | --- | --- | --- |
| Contraceptive Discontinuation | Binary | Yes |  |  |
| Range of services available | Binary | Yes |  | Item (variable) loading on Reports problem w/ availability |
| Availability of medicines | Binary | Yes |  | Item (variable) loading on Reports problem w/ availability |
| Availability of contraceptives | Binary | Yes |  | Item (variable) loading on Reports problem w/ availability |
| Visual privacy during examination | Binary | Yes |  | Item (variable) loading on Reports problem w/ privacy |
| Auditory privacy during examination | Binary | Yes |  | Item (variable) loading on Reports problem w/ privacy |
| Hours of Service | Binary | Yes |  | Item (variable) loading on Reports problem w/ convenience |
| Days of service | Binary | Yes |  | Item (variable) loading on Reports problem w/ convenience |
| Time waited to see a provider | Binary | Yes |  | Item (variable) loading on Reports problem w/ convenience |
| Cleanliness of the facility | Binary | Yes |  | Item (variable) loading on Reports problem w/ clean/staff treatment |
| Staff treatment | Binary | Yes |  | Item (variable) loading on Reports problem w/ clean/staff treatment |
| Amount of explanation received | Binary | Yes |  | Item (variable) loading on Reports problem w/ clean/staff treatment |
| Cost of services or treatment | Binary | Yes |  | Item (variable) loading on Reports problem w/ clean/staff treatment |
| Reports problem w/ availability | Binary | Yes |  |  |
| Reports problem w/ privacy | Binary | Yes |  |  |
| Reports problem w/ convenience | Binary | Yes |  |  |
| Reports problem w/ clean/staff treatment | Binary | Yes |  |  |
| Contraceptive stocks | Binary |  | Yes |  |
| Fee for FP methods | Binary |  | Yes |  |
| Provider discussed FP | Binary | Yes |  |  |
| Used the baseline method before the visit | Binary | Yes |  |  |
| Baseline method | Binary | Yes |  |  |
| Heard FP in radio or TV (last 3 months) | Binary | Yes |  |  |
| CHW talked about FP recently | Binary | Yes |  |  |
| Marital status | Binary | Yes |  |  |
| Age | Binary | Yes |  |  |
| Parity | Categorical | Yes |  |  |
| Education | Categorical | Yes |  |  |
| Wealth | Categorical | Yes |  |  |
| Discussed FP w/ partner (last 6 months) | Binary | Yes |  |  |
